# Combined effects of nucleotide-binding domain-like receptor protein 3 polymorphisms and environmental metals exposure on chronic kidney disease

**DOI:** 10.1038/s41598-022-10098-y

**Published:** 2022-04-15

**Authors:** Yu-Mei Hsueh, Wei-Jen Chen, Ying-Chin Lin, Ya-Li Huang, Horng-Sheng Shiue, Yuh-Feng Lin, Ru-Lan Hsieh, Hsi-Hsien Chen

**Affiliations:** 1 Department of Family Medicine, Wan Fang Hospital, Taipei Medical University, Taipei, Taiwan; 2 Department of Public Health, School of Medicine, College of Medicine, Taipei Medical University, Taipei, Taiwan; 3 Department of Medicine, Section of Epidemiology and Population Sciences, Baylor College of Medicine, Houston, TX USA; 4 Department of Family Medicine, School of Medicine, College of Medicine, Taipei Medical University, Taipei, Taiwan; 5 Department of Geriatric Medicine, School of Medicine, College of Medicine, Taipei Medical University, Taipei, Taiwan; 6 Department of Chinese Medicine, College of Medicine, Chang Gung University, Taoyuan, Taiwan; 7 Graduate Institute of Clinical Medicine, School of Medicine, College of Medicine, Taipei Medical University, Taipei, Taiwan; 8 Division of Nephrology, Department of Internal Medicine, Shuang Ho Hospital, New Taipei, Taiwan; 9 Department of Physical Medicine and Rehabilitation, Shin Kong Wu Ho-Su Memorial Hospital, Taipei, Taiwan; 10 Department of Physical Medicine and Rehabilitation, School of Medicine, College of Medicine, Taipei Medical University, Taipei, Taiwan; 11 Division of Nephrology, Department of Internal Medicine, School of Medicine, College of Medicine, Taipei Medical University, Taipei, Taiwan; 12 Division of Nephrology, Department of Internal Medicine, Taipei Medical University Hospital, Taipei, Taiwan

**Keywords:** Risk factors, Kidney diseases, Environmental impact, Genetic association study, Genotype, Haplotypes

## Abstract

Chronic inflammation is the cause of chronic kidney disease (CKD). The nucleotide-binding domain-like receptor protein 3 (NLRP3) inflammasome plays a vital role in the inflammation process and is associated with the regulatory effects of *NLRP3* gene polymorphisms. This study evaluated the association between *NLRP3* gene polymorphisms and CKD, and further explored whether the association of environmental metals with CKD varied by the *NLRP3* genotypes. A total of 218 CKD patients and 427 age- and sex-matched healthy controls were recruited in this clinic-based case–control study. Patients were identified as having CKD if their estimated glomerular filtration rate (eGFR) < 60 mL/min/1.73 m^2^ and stage 3–5 for at least 3 months. We examined the genotypes of fifteen common ssingle-nucleotide polymorphisms in *NLRP3* genes. Concentrations of total urinary arsenic were examined by summing of urinary inorganic arsenic species. Concentrations of selenium, cadmium, and lead were measured from blood samples. Associations between *NLRP3* polymorphisms, environmental metals exposure, and CKD were evaluated using multivariable logistic regression while controlling for confounders. We observed that the odds of carrying *NLRP3* rs4925650 GA/AA genotypes, *NLRP3* rs1539019 CA/AA genotypes, and *NLRP3* rs10157379 CT/TT genotypes were significantly higher among CKD cases compared to controls, with the adjusted odds ratio (95% confidence interval) were 1.54 (1.01–2.36), 1.56 (1.04–2.33), and 1.59 (1.05–2.38), respectively. The significant multiplicative interactions were identified between high levels of blood lead and *NLRP3* rs4925650 GA/AA genotypes; high levels of blood cadmium or low levels of plasma selenium and the *NLRP3* haplotype (rs4925648, rs4925650, rs12048215, and rs10754555) C-A-A-C multiplicatively interacted to increase the risk of CKD. Our results imply that *NLRP3* polymorphisms may play an important role in the development of environmental metals exposure related CKD.

## Introduction

Chronic kidney disease (CKD) affects 8–16% of the world’s population^1^, resulting in CKD being a common public health problem worldwide^2^. Using estimated glomerular filtration rate (eGFR) < 60 mL/min/1.73 m^2^ to define CKD, the prevalence of CKD in Taiwan was 11.9%, and only 3.5% of patients aware their disease^3^. As the incidence of end-stage renal disease in Taiwan (426/10^6^ in 2012) was the highest globally^4^, CKD is an extremely important health issue in Taiwan.

Our previous study has reported a significantly increased odds of high total urinary arsenic levels and low plasma lycopene levels among the CKD cases compared to the health controls^5^. We also found an increased odds of high blood cadmium and lead levels among CKD cases compared to the controls, whereas a decreased odds of high plasma selenium levels were observed among CKD cases compared to the controls^6^. Recently, a study has shown that plasma concentrations of arsenic and lead were significantly related to the decline of renal function^7^. In addition, a study reported that increasing concentrations of blood cadmium and lead were associated with an increased risk of proteinuria and related to decreased eGFR^8^. These studies suggested that low levels of selenium and high levels of arsenic, cadmium, and lead may increase the risk of CKD. However, the mechanism underlying the effect of these metals on CKD remains unclear.

Inflammation is the cause of several kinds of kidney diseases, such as acute kidney injury and CKD^9^. The nucleotide-binding domain-like receptors 3 (NLRP3) inflammasome is a multi-protein complex that plays an important role in the inflammation process^10^. A previous study has indicated that NLRP3-induced inflammation may promote kidney inflammation and causes CKD^11^. Also, a study has reported that the NLRP3 inflammasome may be involved in the pathogenesis of acute kidney injury, CKD, diabetic nephropathy, and crystal-related nephropathy^12^. Recent evidence has been built to implicate arsenic, cadmium, lead, and selenium on NLRP3 related inflammatory. A study has reported that arsenic activates the NLRP3 inflammasome and induces inflammatory cell death^13^. However, another study showed that arsenic inhibits the secretion of interleukin (IL)-1β and IL-18, which was caused by activating the NLRP3 inflammasomes in macrophages; thus, indicating that exposure to arsenic may affect inflammasome-mediated inflammation^14^. These inconsistent findings reveal an unclear relationship between arsenic exposure and NLRP3 inflammasomes. In addition to arsenic exposure, a carp experiment showed that cadmium exposure induced apoptosis of the anterior spleen and splenic lymphocytes by activating NLRP3^15^. Moreover, lead may activate the NLRP3 signaling pathway to cause oxidative stress and inflammation in chicken testicles, thereby reducing testicular function^16^. In contrast, selenium has a mitigating effect by modifying activation of the NLRP3 signaling pathway in chicken testes caused by lead^16^.

Variations in the *NLRP3* gene may affect mRNA stability and NLRP3 performance^17^. The *NLRP3* gene has nine exons within its 32.9 kb sequence^17^ and is located on chromosome 1q44^18^. There are 60 common single nucleotide polymorphisms (SNPs) that have been identified in the *NLRP3* gene^19^. Several studies have suggested that *NLRP3* polymorphisms were associated with the risk of cardiovascular diseases. A study has found that the 50-year-old subjects with *NLRP3* rs7512998 CC or CT genotypes had a higher blood pressure levels compared to those with TT genotype^20^. In Chinese Han population, *NLRP3* rs10754556 CC genotype was associated with the occurrence of coronary artery disease compared to those with CG or GG genotypes^21^. The *NLRP3* rs4612666 T allele is associated with an increased risk of aorta sclerosis-like ischemic stroke^22^. To date, few studies have explored the association between *NLRP3* gene polymorphisms and CKD. The present study aimed to examine the association between *NLRP3* genotypes and CKD, and to further explore whether *NLRP3* gene polymorphisms may modify the associations of total urinary arsenic, blood cadmium and lead, and plasma selenium with CKD.

## Materials and methods

### Study subjects

This study was a clinic-based case–control study. In total, 218 clinically confirmed CKD patients and 427 age- and sex-matched controls who volunteered to participate in this study were recruited previously^23^. All participants measured serum creatinine concentrations by isotope dilution mass spectrometry (IDMS). The eGFR (mL/min/1.73 m^2^) was calculated using the equation: 186.3 × (serum creatinine)^−1.154^ × (age)^−0.203^ × (0.742 for females)^24^. CKD patients were clinically diagnosed at the Department of Internal Medicine/Nephrology, Taipei Medical University Hospital and Taipei Municipal Wan Fang Hospital, with an eGFR < 60 mL/min/1.73 m^2^ for at least 3 months (stages 3–5) and without hemodialysis according to KDIGO 2012 Clinical Practice Guideline for the Evaluation and Management of Chronic Kidney Disease^25^. Matched controls, with no CKD diagnosis, were recruited from receiving adult and senior citizen health examinations in the Department of Family Medicine. The Research Ethics Committee of Taipei Medical University approved this study (TMU-Joint Institutional Review Board, N201812007) which was conducted in accordance with the Declaration of Helsinki. All participants provided informed consent before the questionnaire interview and specimens collection.

### Interview and specimen collection

The questionnaire interview and the urine and blood samples collection were described previously^5,23^. We measured the arsenic species concentrations in single spot urine samples. EDTA-vacuum syringes were used to collect 5–8 mL peripheral blood samples, and the plasma, red blood cell, and buffy coat were separated. Buffy coat was separated for DNA extraction and to determine *NLRP3* polymorphisms. We analyzed the cadmium and lead concentrations from red blood cells, and measured selenium concentrations from plasma.

### Environmental metals exposure measurement

Urinary concentrations of inorganic-related arsenic species, including trivalent arsenite (As^III^), pentavalent arsenate (As^V^), monomethylarsonic acid (MMA^V^), and dimethylarsinic acid (DMA^V^) were measured as described previously^26^. We assessed total urinary arsenic concentrations by summing the urinary concentrations of As^III^, As^V^, MMA^V^, and DMA^V^. The measure of arsenic in urine specimens was a direct method of excluding nontoxic organic arsenic that contributed to total arsenic exposure^27^. Urinary concentrations were adjusted for urinary creatinine concentrations due to variations in the hydration state^28^. In addition, concentrations of blood cadmium and lead and plasma selenium were determined as described previously^6^. The validity and reliability of the environmental metals exposure measurement are shown in Supplementary Table S1.

### Determination of the genetic polymorphisms

DNA was extracted by digestion with proteinase K followed by phenol and chloroform. We selected 15 common *NLRP3* SNPs based on their minor-allele frequencies (≥ 0.2) in the Han Chinese in the Beijing HapMap database. The Agena Bioscience MassARRAY iPLEX system was used according to the manufacturer’s instructions (National Genome Medicine Center, Taipei, Taiwan) to determine the genotypes of 15 SNPs, including rs4925654, rs4925650, rs12239046, rs4925648, rs10925025, rs10925019, rs1539019, rs3806265, rs10925026, rs10157379, rs12143966, rs10754555, rs3806268, rs12048215, and rs12137901. Among them, rs12137901 did not meet the Hardy–Weinberg equilibrium and was excluded from the statistical analysis. Linkage disequilibrium (LD) strength was determined by calculating D′ and r^2^ of the Lewontin using Haploview 4.1 software^29^.

### Statistical analysis

Differences in the continuous variables were compared between the two groups using the Wilcoxon rank-sum test. The Kruskal–Wallis test was used to compare continuous variables of more than two groups. Multiple logistic regression models were performed estimating odds ratios (ORs) and 95% confidence intervals (CIs) to evaluate the associations between *NLRP3* polymorphism, environmental metal exposure, and CKD while adjusting for confounders. The measures of metal concentrations were categorized into three groups based on the tertile distribution of concentrations among controls. We further treated each tertile group as an ordinal variable in the models to conduct the trend test. Additionally, multiple linear regression models were used to assess associations between *NLRP3* polymorphisms and eGFR while adjusting for confounders. Confounding was informed by prior knowledge and met the criterion that changed the ORs of exposure variables at least by 10% when adding to models assessing between environmental metal exposure and CKD^5^. We explored the interaction by estimating the combined effects of environmental metals exposure (median of concentrations among controls as a cutoff point) and *NLRP3* genotypes on CKD. The product term was added to the logistic regression models to conduct for the multiplicative interaction between metal and *NLRP3* genotypes. The SAS 9.4 (SAS Institute, Cary, NC, USA) was used for statistical analyses. A two-sided *p* value < 0.05 was considered statistically significant.

## Results

The sociodemographic characteristics, eGFR, lifestyle, and disease histories of diabetes and hypertension between CKD cases and controls are shown in Table 1. The mean and standard deviation of age and eGFR in the 218 CKD patients and 427 controls were 65.11 ± 13.52 and 64.21 ± 12.49 years, and 31.54 ± 14.57 and 84.21 ± 15.62 mL/min/1.73 m^2^ respectively. The CKD cases had a lower level of education. Most cases were less likely to have habits of drinking coffee, tea, and alcohol than controls. The CKD patients had significantly increased odds of regularly used analgesics compared to controls, with OR (95% CI) = 2.94 (1.58–5.44). The odds of having disease histories of diabetes and hypertension were 3–5-fold increase among cases compared to controls.

**Table 1:** Sociodemographic characteristics, lifestyle and disease histories, and eGFR between the CKD cases and controls, and the ORs of these variables for CKD.

Variables	CKD cases (N = 218)	Controls (N = 427)	Age-sex adjusted OR (95% CI)
**Sex**			
Male	133 (61.01)	263 (61.59)	1.00
Female	85 (38.99)	164 (38.41)	1.04 (0.74–1.46)^a^
Age	65.11 ± 13.52	64.22 ± 12.49	1.01 (0.99–1.02)^b^
eGFR (mL/min/1.73m^**2**^**)**	31.54 ± 14.57	84.21 ± 15.62	0.35 (0.20–0.60)***
**Educational level**			
Illiterate/elementary school	90 (41.28)	97 (22.83)	1.00^§^
Junior/senior high school	72 (32.03)	150 (35.13)	0.49 (0.33–0.74)***
College and above	56 (25.69)	180 (42.15)	0.31 (0.20–0.48)***
**Cigarette smoking**			
Non-smoker	160 (73.39)	311 (72.83)	1.00
Former smoker	33 (15.14)	74 (17.33)	0.85 (0.53–1.39)
Current smoker	25 (11.47)	42 (9.84)	1.21 (0.69–2.12)
**Alcohol consumption**			
Never	179 (82.11)	274 (64.17)	1.00
Occasional or frequently	39 (17.89)	153 (35.83)	0.36 (0.24–0.55)***
**Coffee consumption**			
Never	170 (77.98)	218 (51.05)	1.00
Occasional or frequently	48 (22.02)	209 (48.95)	0.29 (0.20–0.43)***
**Tea consumption**			
Never	123 (56.42)	149 (34.89)	1.00
Occasional or frequently	95 (43.58)	278 (65.11)	0.41 (0.29–0.58)***
**Analgesic usage**			
No/yes as-needed basis	192 (88.07)	408 (95.55)	1.00
Yes, routinely	26 (11.93)	19 (4.45)	2.94 (1.58–5.44)***
**Diabetes**			
No	132 (60.55)	383 (89.70)	1.00
Yes	86 (39.45)	44 (10.30)	5.71 (3.77–8.66)***
**Hypertension**			
No	94 (43.12)	298 (69.79)	1.00
Yes	124 (56.88)	129 (30.21)	3.14 (2.22–4.44)***

Values are expressed as mean ± standard deviation or number (%) of cases and controls.

*CKD* chronic kidney disease, *eGFR* estimated glomerular filtration rate, *OR* odds ratio, *CI* confidence interval.

***p < 0.001.

^§^*p* < 0.05 for the trend test.

^a^Age adjusted OR and 95% CI.

^b^Sex adjusted OR and 95% CI.

Table 2 presents the association between 14 *NLRP3* gene polymorphisms and CKD. We observed that the odds of carrying *NLRP3* rs4925650 GA/AA genotypes were 1.54-fold (95% CI 1.15–3.34) increased among CKD cases compared to controls after adjusting for covariates. In addition, participants with CKD had a 1.56–1.79 fold increased odds of carrying *NLRP3* rs12239046 CT/TT, *NLRP3* rs10925025 AG/GG, *NLRP3* rs1539019 CA/AA, *NLRP3* rs10925026 CA/AA, and *NLRP3* rs10157379 CT/TT genotypes compared to controls. After additionally adjusting environmental metals exposure, we observed that *NLRP3* rs4925650 [GA/AA vs. GG, OR (95% CI) = 1.89 (0.98–3.35)], *NLRP3* rs1539019 [CA/AA vs. CC, OR (95% CI) = 1.52 (0.95–2.44)], and *NLRP3* rs10157379 [CT/TT vs. CC, OR (95% CI) = 1.50 (0.94–2.41)] were associated with CKD though the confidence interval marginally included the null value. No association was found between other *NLRP3* genotypes and CKD after adjusting for covariates and metals concentrations. *NLRP3* rs4925650, *NLRP3* rs1539019, and *NLRP3* rs10157379 genotypes were selected for further analyzed the effect modification of gene and metal on CKD.

**Table 2:** The association between *NLRP3* gene polymorphisms and CKD.

*NLRP3* genotypes	CKD ases	Controls	Age-sex adjusted ORs (95% CI)	Multivariate adjusted ORs (95% CI)^a^
**rs4925654 G>A**				
GG	159 (73.27)	303 (71.13)	1.00	1.00^§^
GA	54 (24.88)	113 (26.53)	0.90 (0.62–1.32)	0.72 (0.46–1.14)
AA	4 (1.84)	10 (2.35)	0.75 (0.23–2.43)	0.36 (0.08–1.51)
GA/AA versus GG	58 (26.73)	123 (28.87)	0.88 (0.62–1.25)	0.70 (0.45–1.08)
**rs4925650 G>A**				
GG	59 (27.06)	133 (31.44)	1.00^§^	1.00^§^
GA	101 (48.52)	210 (49.65)	1.08 (0.73–1.59)	1.37 (0.87–2.16)
AA	58 (21.53)	80 (18.91)	1.62 (1.02–2.56)*	1.96 (1.15–3.34)*
GA/AA versus GG	159 (72.94)	290 (68.56)	1.23 (0.85–1.77)	1.54 (1.01–2.36)*
**rs12239046 C>T**				
CC	70 (32.11)	166 (38.88)	1.00	1.00
CT	114 (52.29)	189 (44.26)	1.45 (1.01–2.09)*	1.77 (1.16–2.73)**
TT	34 (15.60)	72 (18.86)	1.12 (0.68–1.84)	1.10 (0.62–1.96)
CT/TT versus CC	148 (67.89)	261 (61.12)	1.36 (0.96–1.92)^+^	1.56 (1.04–2.34)*
**rs4925648 C>T**				
CC	124 (56.88)	234 (54.80)	1.00	1.00
CT	78 (35.78)	162 (37.94)	0.92 (0.65–1.30)	0.94 (0.62–1.41)
TT	16 (7.34)	31 (7.26)	0.98 (0.52–1.87)	0.98 (0.47–2.04)
CT/TT versus CC	94 (43.12)	193 (45.20)	0.93 (0.67–1.29)	0.95 (0.64–1.39)
**rs10925025 G>A**				
AA	70 (31.94)	165 (38.73)	1.00	1.00
AG	113 (52.07)	189 (44.37)	1.43 (0.99–2.06)^+^	1.77 (1.15–2.72)**
GG	34 (15.67)	72 (16.90)	1.12 (0.68–1.83)	1.10 (0.62–1.95)
AG/GG versus AA	147 (67.74)	261 (61.27)	1.34 (0.95–1.90)^+^	1.56 (1.04–2.33)*
**rs10925019 C>T**				
CC	102 (46.79)	199 (46.60)	1.00	1.00
CT	101 (46.33)	182 (42.62)	1.09 (0.77–1.53)	0.98 (0.65–1.46)
TT	15 (6.89)	46 (10.77)	0.64 (0.34–1.20)	0.78 (0.38–1.61)
CC/CT versus TT	203 (93.12)	381 (89.23)	0.62 (0.34–1.13)	0.79 (0.40–1.59)
**rs1539019 C>A**				
CC	72 (33.03)	166 (39.06)	1.00	1.00
CA	113 (51.83)	189 (44.47)	1.40 (0.97–2.01)^+^	1.76 (1.15–2.71)**
AA	33 (15.14)	70 (16.47)	1.09 (0.66–1.79)	1.09 (0.61–1.96)
CA/AA versus CC	146 (66.97)	259 (60.94)	1.31 (0.93–1.85)	1.56 (1.04–2.33)*
**rs3806265 T>C**				
TT	68 (31.19)	111 (26.06)	1.00	1.00
TC	108 (49.54)	215 (50.47)	0.79 (0.51–1.24)	0.79 (0.51–1.24)
CC	42 (19.27)	100 (23.47)	0.70 (0.41–1.19)	0.70 (0.41–1.19)
TC/CC versus TT	150 (68.81)	315 (73.94)	0.76 (0.50–1.16)	0.76 (0.50–1.16)
**rs10925026 A>C**				
CC	70 (32.11)	166 (39.06)	1.00	1.00
CA	114 (52.29)	187 (44.00)	0.83 (0.56–1.21)	1.79 (1.16–2.74)**
AA	34 (15.60)	72 (16.94)	0.69 (0.43–1.11)	1.10 (0.62–1.95)
CA/AA versus CC	148 (68.35)	259 (60.94)	0.79 (0.55–1.13)	1.57 (1.05–2.35)*
**rs10157379 T>C**				
CC	69 (31.65)	164 (38.68)	1.00	1.00
CT	114 (52.29)	189 (44.58)	1.45 (1.01–2.10)*	1.79 (1.16–2.75)**
TT	35 (16.06)	71 (16.75)	1.18 (0.72–1.93)	1.15 (0.65–2.04)
CT/TT versus CC	149 (68.35)	259 (60.94)	1.38 (0.97–1.95)^+^	1.59 (1.05–2.38)*
**rs12143966 A>G**				
GG	57 (26.15)	125 (29.83)	1.00	1.00
GA	112 (51.38)	191 (45.58)	1.29 (0.87–1.95)	1.41 (0.89–2.21)
AA	49 (22.48)	103 (24.58)	1.04 (0.66–1.66)	1.14 (0.67–1.96)
GA/AA versus GG	161 (73.85)	294 (70.17)	1.20 (0.83–1.74)	1.31 (0.86–2.01)
**rs10754555 C>G**				
CC	85 (38.99)	154 (36.15)	1.00	1.00
CG	102 (46.79)	199 (46.71)	0.93 (0.65–1.35)	0.87 (0.57–1.32)
GG	31 (14.22)	73 (17.14)	0.78 (0.47–1.28)	0.69 (0.39–1.23)
CG/GG versus CC	133 (61.01)	271 (63.85)	0.89 (0.64–1.15)	0.82 (0.55–1.21)
**rs3806268 A>G**				
AA	68 (31.19)	113 (26.59)	1.00	1.00
AG	108 (49.54)	212 (49.88)	0.85 (0.58–1.25)	0.84 (0.54–1.31)
GG	42 (19.27)	100 (23.53)	0.71 (0.44–1.13)	0.73 (0.43–1.26)
AG/GG versus AA	150 (68.81)	312 (73.41)	0.81 (0.56–1.15)	0.80 (0.53–1.22)
**rs12048215 A>G**				
AA	103 (47.25)	192 (44.96)	1.00	1.00
AG	93 (42.66)	185 (43.33)	0.94 (0.67–1.33)	0.93 (0.62–1.41)
GG	22 (10.09)	50 (11.71)	0.83 (0.47–1.44)	0.95 (0.50–1.77)
AG/GG versus AA	115 (52.75)	235 (55.02)	0.92 (0.66–1.28)	0.94 (0.64–1.38)

*NLRP3* rs3806265 and rs10754555 were missing for one participant. *NLRP3* rs4925654, rs10925025, rs1539019, rs10925026, and rs3806268 were missing for two participants. *NLRP3* rs10157379 was missing for three participants. *NLRP3* rs4925650 was missing for four participants. *NLRP3* rs12143966 was missing for nine participants.

*CKD* chronic kidney disease, *NLRP3* nucleotide-binding domain-like receptors 3, *OR* odds ratio, *CI* confidence interval.

^+^0.05 ≤ *p* < 0.1, *p < 0.05, **p < 0.01.

^§^*p* < 0.05 for the trend test.

^a^Adjusted for age, sex, educational level, alcohol, coffee and tea consumption, analgesic usage, and disease histories of diabetes and hypertension.

LD and haplotype analyses revealed that the *NLRP3* genes exhibited the four haplotype blocks shown in Supplementary Figure S1. D’ of Lewontin of the haplotype *NLRP3* block 1 (*NLRP3* rs4925648, *NLRP3* rs4925650, *NLRP3* rs12048215, and *NLRP3* rs10754555), *NLRP3* block 2 (*NLRP3* rs3806265 and *NLRP3* rs38062628), *NLRP3* block 3 (*NLRP3* rs10925019 and *NLRP3* rs4925654), and *NLRP3* block 4 (*NLRP3* rs1539019, *NLRP3* rs10925025, *NLRP3* rs12143966, *NLRP3* rs12239046, *NLRP3* rs10925026, and *NLRP3* rs10157379) ranged from 0.99 to 1.00. The association between the *NLRP3* gene haplotypes and CKD is shown in Table 3. We observed that the odds of C-G-A-C and C-G-A-G haplotypes in the *NLRP3* block 1 respectively decreased by 47% and 52% among the CKD cases compared to the controls. In addition, the combined T-G-G-G, C-G-A-C, C-G-A-G, and C-G-G-G haplotypes in the *NLRP3* block 1 were significantly inversely associated with CKD compared to the C-A-A-C haplotype. These associations remained statistically significant after further adjusting for metals concentrations in the models. The odds of C-A haplotype in *NLRP3* block 3 were decreased by 35% among the CKD cases compared to the controls. No association was observed between *NLRP3* blocks 2 and 4 and CKD.

**Table 3:** The association between *NLRP3* gene haplotypes and CKD.

*NLRP3* haplotypes	CKD Cases	Controls		Age-sex adjusted ORs (95% CI)	Multivariate adjusted ORs (95% CI)^a^
***NLRP3* block 1: rs4925648, rs4925650, rs12048215, and rs10754555**					
C-A-A-C	215 (49.65)	370 (43.94)		1.00	1.00
T-G-G-G	108 (24.94)	224 (26.60)		0.84 (0.63–1.11)	0.79 (0.57–1.10)
C-G-A-C	56 (12.93)	133 (15.80)		0.73 (0.51–1.04)^+^	0.63 (0.42–0.95)*
C-G-A-G	27 (6.24)	62 (7.36)		0.75 (0.46–1.22)	0.48 (0.27–0.85)*
C-G-G-G	27 (6.24)	53 (6.29)		0.79 (0.49–1.28)	0.82 (0.47–1.43)
T-G-G-G/C-G-A-C/C-G-A-G/C-G-G-G versus C-A-A-C	218 (50.35)	472 (56.06)		0.79 (0.63–0.99)*	0.70 (0.54–0.92)*
***NLRP3* block 2: rs3806265 and rs3806268**					
T-A	242 (55.50)	437 (51.41)		1.00	1.00
C-G	190 (43.58)	411 (48.35)		0.84 (0.67–1.06)	0.85 (0.64–1.11)
C-A	3 (0.69)	1 (0.12)		0.61 (0.06–5.93)	0.35 (0.03–4.70)
T-G	1 (0.23)	1 (0.12)		1.67 (0.10–27.00)	3.72 (0.23–61.18)
C-G/C-A/T-G versus T-A	192 (44.04)	413 (48.59)		0.84 (0.67–1.06)	0.85 (0.65–1.11)
***NLRP3* block 3: rs10925019 and rs4925654**					
C-G	242 (55.76)		445 (52.11)	1.00	1.00
T-G	130 (29.95)		275 (32.20)	0.88 (0.67–1.14)	0.85 (0.62–1.14)
C-A	62 (14.29)		134 (15.69)	0.85 (0.60–1.19)	0.65 (0.43–0.97)*
T-G/C-A versus C-G	192 (44.24)		409 (47.89)	0.87 (0.69–1.09)	0.78 (0.59–1.02)^+^
***NLRP3* block 4: rs1539019, rs10925025, rs12143966, rs12239046, rs10925026, and rs10157379**					
C-G-A-C-A-T	222 (51.39)		440 (52.38)	1.00	1.00
A-A-G-T-C–C	177 (40.97)		322 (38.33)	1.09 (0.85–1.39)	1.15 (0.87–1.53)
C-G-G-C-A-T	27 (6.25)		72 (8.57)	0.74 (0.46–1.18)	0.85 (0.43–1.49)
C-A-G-T-C–C	4 (0.93)		3 (0.36)	2.71 (0.60–12.24)	1.47 (0.26–8.34)
C-G-A-C-A-C	1 (0.23)		2 (0.24)	0.97 (0.09–10.79)	1.25 (0.11–14.52)
A-G-A-C-A-T	0		1 (0.12)	–	–
A-G-G-C-A-T	1 (0.23)		0	–	–
A-A-G-T-C–C/C-G-G-C-A-T/C-A-G-T-C–C/C-G-A-C-A-C/A-G-A-C-A-T/A-G-G-C-A-T versus C-G-A-C-A-T	210 (48.61)		400 (47.62)	1.04 (0.82–1.31)	1.11 (0.85–1.45)

*CKD* chronic kidney disease, *NLRP3* nucleotide-binding domain-like receptors 3, *OR* odds ratio, *CI* confidence interval.

^+^0.05 ≤ *p* < 0.1, *p < 0.05.

^a^Adjusted for age, sex, educational level, alcohol, coffee and tea consumption, analgesic usage, and disease histories of diabetes and hypertension.

Associations between *NLRP3* rs4925650, *NLRP3* rs1539019, and *NLRP3* rs10157379 genotypes and eGFR are shown in Table 4. Participants who carried the *NLRP3* rs4925650 AA genotype decreased 7.62 mL/min/1.73 m^2^ of eGFR when compared to those carrying the GG genotype. Participants who carried the *NLRP3* rs1539019 A allele and the *NLRP3* rs10157379 T allele decreased eGFR by 6 mL/min/1.73 m^2^ when compared with those carrying the *NLRP3* rs1539019 C allele and the *NLRP3* rs10157379 C allele. These results showed no changes after adjusting for blood cadmium and lead or plasma selenium levels in models.

**Table 4:** The association between *NLRP3* gene polymorphisms and eGFR.

*NLRP3* genotypes	Genotypes/Alleles	β (SE)^a^	*p* values
rs4925650 G>A	GA versus GG	− 1.32 (2.40)	0.582
	AA versus GG	− 7.62 (2.90)	0.009
	GA/AA versus GG	− 3.30 (2.26)	0.145
	A versus G	− 3.06 (2.26)	0.175
rs1539019 C>A	CA versus CC	− 7.47 (2.26)	0.001
	AA versus CC	− 0.54 (3.05)	0.859
	CA/AA versus CC	− 5.68 (2.14)	0.008
	A versus C	− 5.93 (2.14)	0.006
rs10157379 T>C	CT versus CC	− 7.09 (2.27)	0.002
	TT versus CC	− 1.32 (3.03)	0.664
	CT/TT versus CC	− 5.58 (2.14)	0.009
	T versus C	− 5.84 (2.14)	0.007

eGFR, estimated glomerular filtration rate (mL/min/1.73 m^2^); β, Regression coefficient; SE, Standard error of regression coefficient.

^a^Adjusted for age, sex, educational level, alcohol, coffee and tea consumption, analgesic usage, disease histories of diabetes and hypertension, and total urinary arsenic.

Environmental metals exposure was found to be associated with CKD in our study (Supplementary Table S2). As the levels of total urinary arsenic and blood cadmium and lead increased, the OR of CKD increased significantly in a dose–response manner. In contrast, as the levels of plasma selenium increased, the OR of CKD decreased significantly in a dose–response relationship. No difference was observed in comparing concentrations of environmental metals exposure by different genotypes of *NLRP3* rs4925650, *NLRP3* rs1539019, and *NLRP3* rs10157379.

**Figure 1:**
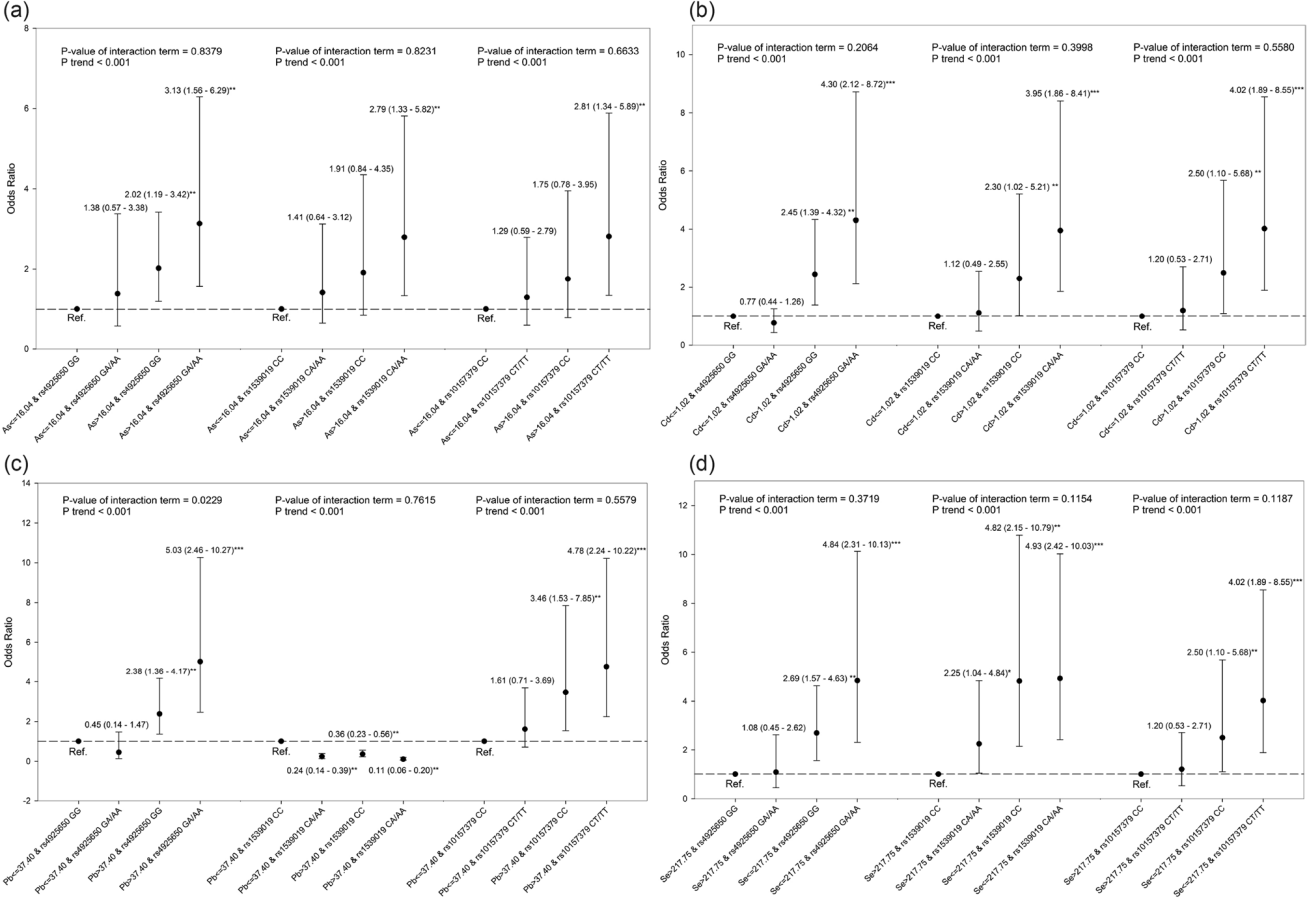
The combined effect of *NLRP3* rs4925650, *NLRP3*rs 1,539,019, *NLRP3* rs10157379, and levels of environmental metals exposure on the CKD. (**a**) Total urinary arsenic; (**b**) Blood cadmium; (**c**) Blood lead; (**d**) Plasma selenium. The estimates of OR were adjusted for age, sex, educational level, alcohol, coffee and tea consumption, analgesic usage, disease histories of diabetes and hypertension, and levels of other metals.

**Figure 2:**
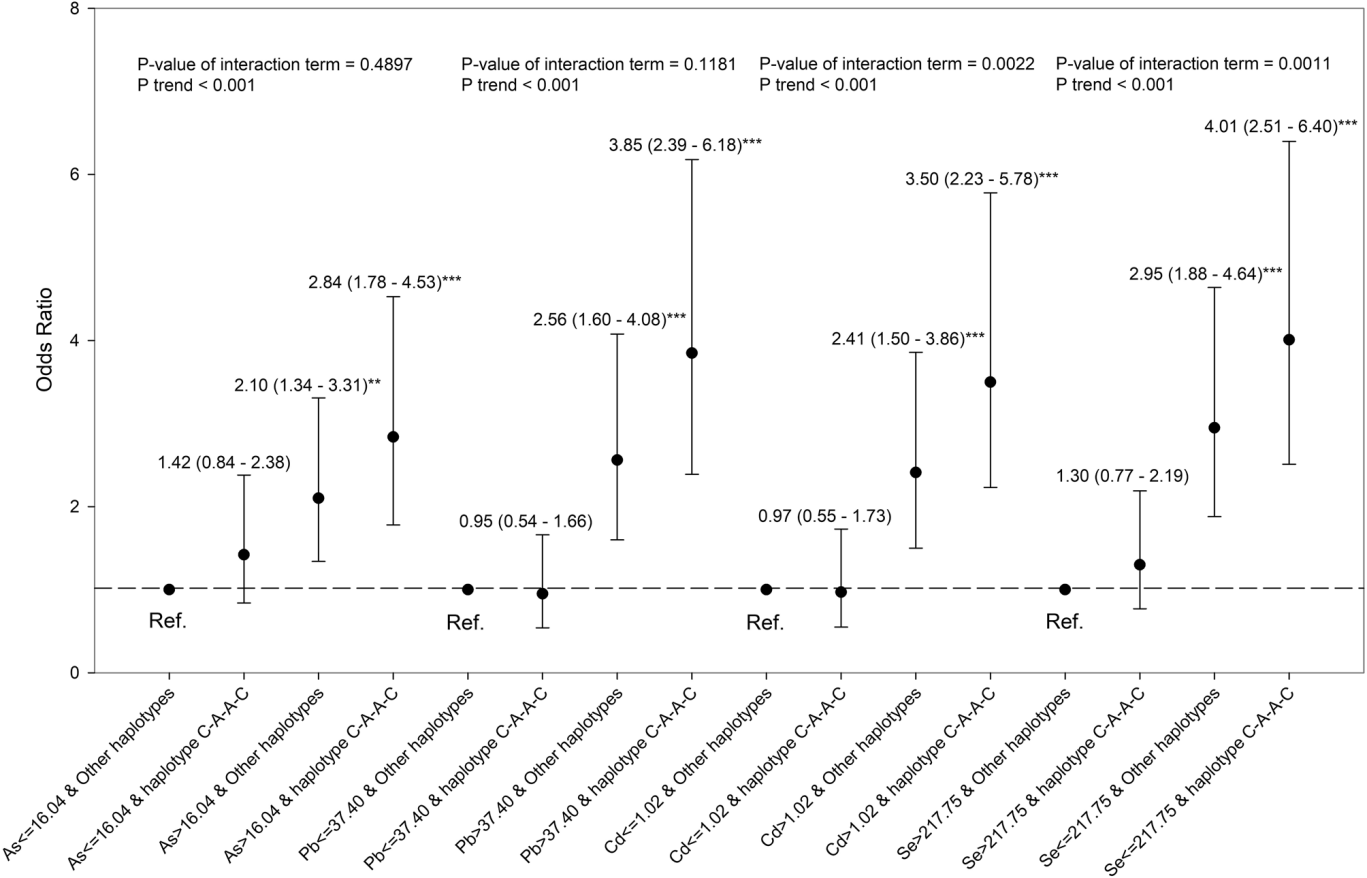
The combined effect of the *NLRP3* block 1 haplotypes (rs4925648, rs4925650, rs12048215, and rs10754555) and levels of total urinary arsenic, blood lead and cadmium, and plasma selenium on CKD. The estimates of OR were adjusted for age, sex, educational level, alcohol, coffee and tea consumption, analgesic usage, disease histories of diabetes and hypertension, and levels of other metals.

Figure 1 shows the combined effect of *NLRP3* rs4925650, *NLRP3* rs1539019, *NLRP3* rs10157379, and levels of environmental metals exposure on the CKD. The trend analysis showed that the OR of CKD increased gradually with exposure to no risk factors, one risk factor, or two risk factors (risk genotypes, high levels of arsenic, cadmium, and lead, or low levels of selenium). We observed that the odds of carrying *NLRP*3 rs4925650 GA/AA genotypes and high levels of blood lead (> 37.40 μg/L) were 5.03-fold increased (95% CI 2.46–10.27) among CKD cases compared to controls (Fig. 1C). The p-value of the interaction term of *NLRP*3 rs4925650 and levels of blood lead was 0.0229, which indicated a multiplicative interaction between *NLRP*3 rs4925650 and blood lead on CKD. In addition, we observed that *NLRP3* block 1 (risk haplotype: C-A-A-C) interacted with total urinary arsenic, blood lead and cadmium, and plasma selenium to significantly enhance the OR of CKD, respectively (Fig. 2). High levels of blood cadmium and the *NLRP3* block 1 C-A-A-C haplotype, and low levels of plasma selenium and the *NLRP3* block 1 C-A-A-C haplotype significantly and multiplicatively interacted to increase the OR of CKD, respectively.

## Discussion

To the best of our knowledge, this study is the first to evaluate the associations between *NLRP3* polymorphisms, environmental metals exposure, and CKD. We found that the odds of carrying *NLRP3* rs4925650 GA/AA genotypes, *NLRP3* rs1539019 CA/AA genotypes, and *NLRP3* rs10157379 CT/TT genotypes were significantly higher among CKD cases compared to controls. In addition, certain *NLRP3* genotypes were interacting with environmental metals exposure on the risk of CKD. Specifically, high levels of blood lead and *NLRP3* rs4925650 GA/AA genotypes; high levels of blood cadmium and *NLRP3* block 1 C-A-A-C haplotype, and low levels of plasma selenium and *NLRP3* block 1 C-A-A-C haplotype significantly and multiplicatively interacted to increase the risk of CKD.

Persistent inflammation and activation of the innate immune system is a chronic kidney damage phenomenon, which is important for the development of CKD^30^. The mechanism of inflammation-induced CKD is still unknown. The pattern recognition receptors, such as nucleotide oligomerization domain-like receptors (NLRs), C-type lectin-like receptors, toll-like receptors, and retinoic acid-inducible gene I-like receptors act as sensors of the innate immune system^31^. The most characteristic inflammasome is NLRP3, which responds to endogenous damage signals caused by entry of pathogen or tissue damage^32^. The NLRP3 inflammasome is believed to play a key role in the underlying inflammatory response in many chronic diseases, including CKD^33^.

Genetic variation in the *NLRP3* gene may be an important determinant of the degree of the immune inflammatory response, which affects susceptibility to inflammatory diseases^34,35^. In the present study, *NLRP3* rs1539019 CA/AA genotypes significantly increased with the risk of CKD after adjusting for confounders. Various studies have evaluated the association of *NLRP3* rs1539019 polymorphism with several health-related outcomes. Patients with chronic hepatitis C virus and the *NLRP3* rs1539019 AA genotype do not respond to interferon therapy^36^, and the *NLRP3* rs1539019 TT genotype is related to pneumoconiosis in Chinese coal workers^37^. In addition, it has been reported that the *NLRP3* rs1539019 A allele is related to circulating fibrinogen concentration and therefore to the risk of cardiovascular disease^38^. The rs1539019 G>T locus is close to a 12-nucleotide sequence identified as a consensus binding site for epidermal growth factor 1 that may influence the vertebrate blood coagulation network^39^. *NLRP3* rs1539019 A allele has been indicated to increase circulating fibrinogen levels^38^, an indicator of inflammation, which may be associated with blood coagulation, followed by leading to CKD. However, another study found no significant association between the *NLRP3*rs1539019 polymorphism and the risk of essential hypertension in a Japanese population^40^. The *NLRP3* rs1539019 polymorphism was not associated with primary gouty arthritis in the Chinese Han population^41^. *NLRP3* rs1539019 is an intronic polymorphism and it is unclear how genetic variations in introns affect gene function. However, one study reported that many transcription factors bind to intron sites, and these intron sites may play a role regulating gene expression^42^.

In addition to *NLRP3* rs1539019 polymorphism, we also found that *NLRP3* rs4925650 GA/AA genotypes and *NLRP3* rs10157379 CT/TT genotypes significantly increased the risk of CKD after adjusting for confounders. Hence, the effect of *NLRP3* rs4925650 GA/AA genotypes and *NLRP3* rs10157379 CT/TT genotypes on susceptibility to CKD appears to be independent of age, sex, educational level, consumption of tea, alcohol, and coffee, analgesic usage, and disease histories of diabetes and hypertension. The present study found an association between the *NLRP3* rs10157379 CT/TT genotypes and CKD in the Taiwanese population, which is in accordance with our previous research showed that the *NLRP3* rs10157379 T allele has a borderline association with renal cell carcinoma^43^. These results imply that subjects with *NLRP3* rs10157379 T allele may be associated with renal damage than those with C allele. In addition, a recent study found that the *NLRP3* rs10157379 CT genotype was associated with the severity of severe acute respiratory syndrome (SARS)^44^. NLRP3 may also be related to host immunity and susceptibility to inflammation disorders^45^ . Further, NLRP3 can interact with thioredoxin-interacting protein (TXNIP), a protein involved in insulin resistance. TXNIP deficiency may impair the activation of the NLRP3 inflammasome and subsequent secretion of interleukin 1β, which was involved in the pathogenesis of diabetes^46^. As diabetes is a recognized risk factor for CKD, *NLRP3* genes may be involved in diabetes-related CKD. Additionally, the haplotype analyses were performed showing that the *NLRP3* block 1 C-G-A-C, C-G-A-G, C-G-G-G, or T-G-G-G haplotypes significantly decreased the OR of CKD compared to that of the C-A-A-C haplotype (which includes the *NLRP3* rs4925650 A allele). To date, epidemiological studies evaluating the effect of *NLRP3* polymorphisms on CKD are limited, further studies are needed to explore the possible mechanism of the associations between these genotypes, the haplotype, and CKD.

Several in vitro and in vivo studies have indicated that metals exposure may affect NLRP3 functional changes. An in vitro study has reported that the insulin resistance induced by NaAsO_2_ is due to activation of the NLRP3 inflammasome^47^. Chicken experiments have shown that the NLRP3 signaling pathway is activated by lead-induced oxidative stress after lead administration, which causes testicular damage^16^. Other animal studies have shown that cadmium chloride induces testicular injury^48^ or liver injury^49^ in mice by activating the NLRP3 signaling pathway. A selenium-rich basal diet may inhibit lipopolysaccharide-induced inflammation in chicken liver by suppressing the toll like receptor 4-nuclear factor kB-NLRP3 signaling pathway^50^. Also, a study has reported that dietary selenium attenuates *Staphylococcus aureus* mastitis in mice by inhibiting the NLRP3 inflammasome^51^. Our study found no difference when comparing concentrations of environmental metals exposure by different genotypes of *NLRP3* rs4925650, *NLRP3* rs1539019, and *NLRP3* rs10157379, which suggests that metals exposure and the *NLRP3* genes have independent effects on CKD. Additional studies are needed to better understand the effect of environmental metals exposure in NLRP3 function and its mechanism.

In the present study, we observed that high levels of blood lead and *NLRP3* rs4925650 GA/AA genotypes significantly interacted to increase the risk of CKD after multivariate adjustment. This may be because lead in the blood can induce alterations of inflammatory marker NLRP3 inflammasome activation^16^, and reduced eGFR^6^, leading to an increase in the risk of CKD. We also found that high levels of blood cadmium and the *NLRP3* block 1 haplotype C-A-A-C multiplicatively interacted to increase the risk of CKD after adjusting for multiple risk factors. Evidence has shown that cadmium in the blood may inhibit heme oxygenase 1 and nuclear factor erythroid 2-related factor 2, and activate the NLRP3 inflammasome^49^ or increase reactive oxygen species to activate the NLRP3 inflammasome^48^, and decreased eGFR^6^, which may jointly cause CKD pathogenesis^52^. In addition, we observed that low plasma selenium level and the *NLRP3* block 1 haplotype C-A-A-C multiplicatively interacted to increase the risk of CKD after adjusting for multiple risk factors. Studies have found that a low level of plasma selenium may not inhibit the expression of NLRP3^51^, or downregulate the toll-like receptor 4-nuclear factor-kB-NLRP3 signaling pathway^50^, which may increase kidney inflammation to increase the risk of CKD^53^. Our study did not precisely measure the levels of serum NLRP3 inflammasome. Therefore, whether the NLRP3 inflammasome was affected by levels of blood cadmium and lead, low levels of plasma selenium or the *NLRP3* polymorphisms remain unknown. The underlying mechanism of joint effect of environmental metals exposure and *NLRP3* polymorphisms affecting CKD needs further exploration.

The sample size of this study was small with limited statistical power. Further studies with larger sample size are needed to improve the precision of point estimates when assessing the effect modification of *NLRP3* gene polymorphisms and environmental metals exposure on CKD. Additionally, unmeasured factors such as blood pressure medication and treatment might have potentially influenced our observed results. Future studies should consider these factors in multiple regression models. The analysis of 15 *NLRP3* gene polymorphisms may not represent those of the entire gene functions. Our study did not analyze gene polymorphisms that regulate NLRP3 expression differently according to the disease conditions. Further studies should be carried out to evaluate the function of NLRP3 and its related gene polymorphisms in order to determine their role in CKD development.

## Conclusions

In conclusion, the present study found evidence that the *NLRP3* rs4925650 GA/AA genotypes or NLRP3 block 1 haplotype C-A-A-C altered the risk of CKD related to high levels of blood lead and cadmium, or low levels of plasma selenium. Future studies are warranted to measure the levels of serum NLRP3 inflammasome, to elucidate the biological mechanism underlying the associations between *NLRP3* polymorphisms, environmental metals exposure, and CKD.

## Supplementary Information

Supplementary Information.
