# Author Correction: Combined effects of nucleotide-binding domain-like receptor protein 3 polymorphisms and environmental metals exposure on chronic kidney disease

**DOI:** 10.1038/s41598-022-27056-3

**Published:** 2022-12-30

**Authors:** Yu‑Mei Hsueh, Wei‑Jen Chen, Ying‑Chin Lin, Ya‑Li Huang, Horng‑Sheng Shiue, Yuh‑Feng Lin, Ru‑Lan Hsieh, Hsi‑Hsien Chen

**Affiliations:** 1 Department of Family Medicine, Wan Fang Hospital, Taipei Medical University, Taipei, Taiwan; 2 Department of Public Health, School of Medicine, College of Medicine, Taipei Medical University, Taipei, Taiwan; 3 Department of Medicine, Section of Epidemiology and Population Sciences, Baylor College of Medicine, Houston, TX USA; 4 Department of Family Medicine, School of Medicine, College of Medicine, Taipei Medical University, Taipei, Taiwan; 5 Department of Geriatric Medicine, School of Medicine, College of Medicine, Taipei Medical University, Taipei, Taiwan; 6 Department of Chinese Medicine, College of Medicine, Chang Gung University, Taoyuan, Taiwan; 7 Graduate Institute of Clinical Medicine, School of Medicine, College of Medicine, Taipei Medical University, Taipei, Taiwan; 8 Division of Nephrology, Department of Internal Medicine, Shuang Ho Hospital, New Taipei, Taiwan; 9 Department of Physical Medicine and Rehabilitation, Shin Kong Wu Ho-Su Memorial Hospital, Taipei, Taiwan; 10 Department of Physical Medicine and Rehabilitation, School of Medicine, College of Medicine, Taipei Medical University, Taipei, Taiwan; 11 Division of Nephrology, Department of Internal Medicine, School of Medicine, College of Medicine, Taipei Medical University, Taipei, Taiwan; 12 Division of Nephrology, Department of Internal Medicine, Taipei Medical University Hospital, Taipei, Taiwan

Correction to: *Scientific Reports* https://doi.org/10.1038/s41598-022-10098-y, published online 15 April 2022

The original version of this Article contained an error in the Materials and methods section, under the subheading ‘Study subjects’, where the TMU-Joint Institutional Review Board assigned number was incorrect.

“The Research Ethics Committee of Taipei Medical University approved this study (TMU-Joint Institutional Review Board, N201912058) which was conducted in accordance with the Declaration of Helsinki.”

now reads:

“The Research Ethics Committee of Taipei Medical University approved this study (TMU-Joint Institutional Review Board, N201812007) which was conducted in accordance with the Declaration of Helsinki.”

The original Article has been corrected.

