# Supplementary material for: Combined effects of nucleotide-binding domain-like receptor protein 3 polymorphisms and environmental metals exposure on chronic kidney disease
Source: Sci Rep. 2022 Apr 15;12:6307. doi: 10.1038/s41598-022-10098-y (PMC9012248; doi:10.1038/s41598-022-10098-y)
Supplement: Supplementary file 1 — Supplementary Information. [file 41598_2022_10098_MOESM1_ESM.docx]

**Supplementary Tables**

Table S1. The validity and reliability of urinary arsenic species, red blood cell cadmium and lead, and plasma selenium.

| **Metals or metalloids** | **Methods** | **Detection limit (μg/L)** | **Recovery rate** | **Standard reference materials (SRM)** | **CV%** |
| --- | --- | --- | --- | --- | --- |
| Arsenite (As^III^) | High performance liquid chromatography-hydride generator-atomic absorption spectrometry | 0.02 | 93.8% to 102.2% | SRM 2670 (National Institute of Standards and Technology, Gaithersburg, MD) certificate value 480 ± 100 μg/L inorganic arsenic, in our system 507 ± 17 μg/L (*n* = 4). | < 10% |
| Arsenate (As^V^) |  | 0.10 |  |  |  |
| Monomethylarsonic acid (MMA^V^) |  | 0.07 |  |  |  |
| Dimethylarsinic acid (DMA^V^) |  | 0.06 |  |  |  |
| Red blood cell cadmium | Inductively coupled plasma mass spectrometry | 0.07 |  | SRMs (Seronorm Trace Elements Whole Blood L-2 (Lot 1103129)) certificate value 5.8 μg/L (range: 5.4–6.2 μg/L) in our system 6.1 ± 0.5 μg/L | < 10% |
| Red blood cell lead | Inductively coupled plasma mass spectrometry | 0.32 |  | SRMs (Seronorm Trace Elements Whole Blood L-2 (Lot 1103129)) certificate value 310.0 μg/L (range 186.0–434.0 μg/L), in our system 329.0 ± 17.0 μg/L | < 10% |
| Plasma selenium | Inductively coupled plasma mass spectrometry | 0.193 |  | SRMs (Seronorm Trace Elements Whole Blood Label II (SERO AS, Norway)) contained 112 ± 46 mg/L of selenium, in our system 118.7 ± 11.1 mg/L (n = 7) | 9.8% |

Table S2. Associations of total urinary arsenic, blood lead and cadmium, and plasma selenium with CKD.

| Variables | CKD cases | Controls | Multivariate ORs  (95% CI)^a^ | Multivariate ORs  (95% CI) |
| --- | --- | --- | --- | --- |
| Total urinary arsenic (μg/g creatinine) | 27.28 ± 21.74 | 19.95 ± 13.91^#^ |  |  |
| ≤ 12.07 | 36 (15.51) | 142 (33.26) | 1.00^§,***^ | 1.00^b, §,***^ |
| 12.07 - 22.00 | 70 (32.11) | 142 (33.26) | 1.98 (1.16-3.36)^*^ | 1.75 (0.96-3.16)^+^ |
| > 22.00 | 112 (51.38) | 143 (33.49) | 3.30 (1.95-5.59)^***^ | 2.77 (1.53-5.03)^***^ |
|  |  |  |  |  |
| Red blood cell cadmium (μg/L) | 2.44 ± 3.46 | 1.22 ± 0.93^#^ |  |  |
| ≤ 0.80 | 20 (9.17) | 144 (33.72) | 1.00^§,***^ | 1.00^c, §,***^ |
| 0.80 - 1.30 | 47 (21.56) | 145 (33.96) | 3.40 (1.73-6.63)^***^ | 2.42 (1.44-5.11)^*^ |
| > 1.30 | 151 (69.27) | 138 (32.32) | 13.14 (6.81-25.36)^**^ | 6.62 (3.15-13.89)^**^ |
|  |  |  |  |  |
| Red blood cell lead (μg/L) | 68.74 ± 38.94 | 42.00 ± 23.00^#^ |  |  |
| ≤ 27.94 | 20 (9.17) | 142 (33.26) | 1.00^§,***^ | 1.00^c,§,***^ |
| 27.94 – 46.35 | 50 (22.94) | 143 (33.49) | 2.98 (1.55-5.74)^**^ | 2.98 (1.45-6.10)^**^ |
| > 46.35 | 148 (67.89) | 142 (33.26) | 9.91 (5.35-18.36)^***^ | 6.08 (3.06-12.11)^***^ |
|  |  |  |  |  |
| Plasma selenium (μg/L) | 193.30 ± 69.81 | 218.60 ± 53.42^#^ |  |  |
| ≤ 196.70 | 135 (61.93) | 143 (33.49) | 1.00^§,***^ | 1.00^c, §,***^ |
| 196.70 -243.90 | 48 (22.02) | 142 (33.26) | 0.23 (0.14-0.38)^***^ | 0.29 (0.16-0.52)^***^ |
| > 243.90 | 35 (16.06) | 142 (33.26) | 0.13 (0.08-0.23)^***^ | 0.21 (0.11-0.39)^***^ |

Abbreviations: CKD, chronic kidney disease; OR, odds ratio; CI, confidence interval.

Values are expressed as mean ± standard deviation or number (%) of cases and controls.

^a^ Adjusted for age, gender, educational level, alcohol, coffee and tea consumption, analgesic usage, and disease histories of diabetes and hypertension.

^b^ Adjusted for age, gender, educational level, alcohol, coffee and tea consumption, analgesic usage, disease histories of diabetes and hypertension, and levels of plasma selenium, blood cadmium and lead.

^c^ Adjusted for age, gender, educational level, alcohol, coffee and tea consumption, analgesic usage, disease histories of diabetes and hypertension, levels of total urinary arsenic, and levels of other metals (plasma selenium, blood lead or cadmium).

^#^ *p* < 0.05 for the Wilcoxon rank-sum test.

^+^ 0.05 ≤ *p* < 0.1, ^*^*p* < 0.05, ^**^*p* < 0.01, ^***^*p* < 0.001.

^§^ *p* < 0.05 for the trend test.

**Supplementary Figure**

| (A) | 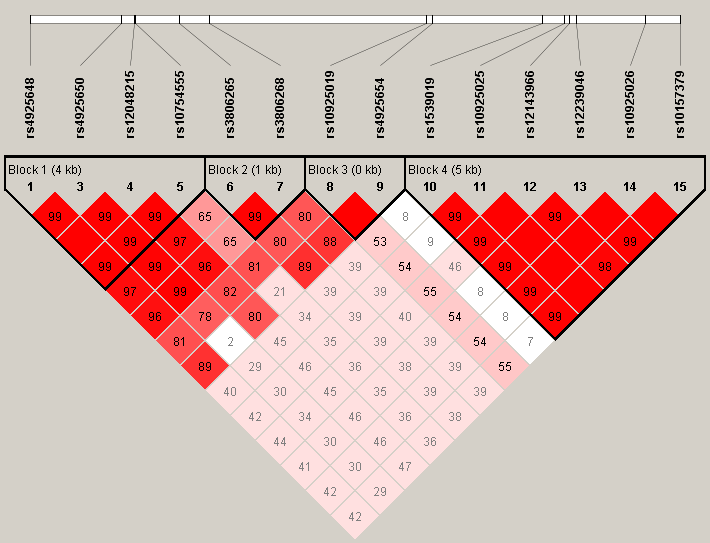 |
| --- | --- |
| (B) | 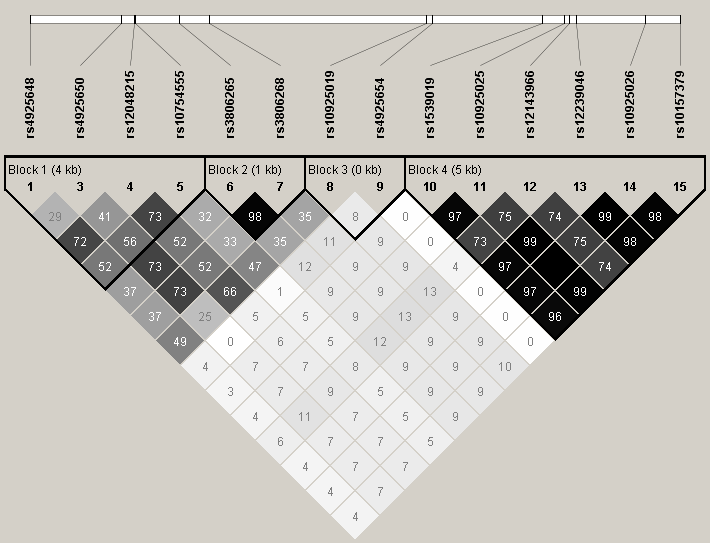 |
| Figure S1. (A) Lewontin’s D’ of the *NLRP3* block 1 (*NLRP3* rs4925648, *NLRP3* rs4925650, *NLRP3* rs12048215, and *NLRP3* rs10754555), *NLRP3* block 2 (*NLRP3* rs3806265 and *NLRP3* rs38062628), *NLRP3* block 3 (*NLRP3* rs10925019 and *NLRP3* rs4925654), and *NLRP3* block 4 (*NLRP3* rs1539019, *NLRP3* rs10925025, *NLRP3* rs12143966, *NLRP3* rs12239046, *NLRP3* rs10925026, and *NLRP3* rs10157379) polymorphisms. (B) *r^2^* values for each pair of polymorphisms of the *NLRP3*. | |
